# Protozoan Predation of *Escherichia coli* O157:H7 Is Unaffected by the Carriage of Shiga Toxin-Encoding Bacteriophages

**DOI:** 10.1371/journal.pone.0147270

**Published:** 2016-01-29

**Authors:** Carrie E. Schmidt, Smriti Shringi, Thomas E. Besser

**Affiliations:** Department of Veterinary Microbiology and Pathology, College of Veterinary Medicine, Washington State University, Pullman, Washington, United States of America; The Pennsylvania State University, UNITED STATES

## Abstract

*Escherichia coli* O157:H7 is a food-borne bacterium that causes hemorrhagic diarrhea and hemolytic uremic syndrome in humans. While cattle are a known source of *E*. *coli* O157:H7 exposure resulting in human infection, environmental reservoirs may also be important sources of infection for both cattle and humans. Bacteriophage-encoded Shiga toxins (Stx) carried by *E*. *coli* O157:H7 may provide a selective advantage for survival of these bacteria in the environment, possibly through their toxic effects on grazing protozoa. To determine Stx effects on protozoan grazing, we co-cultured *Paramecium caudatum*, a common ciliate protozoon in cattle water sources, with multiple strains of Shiga-toxigenic *E*. *coli* O157:H7 and non-Shiga toxigenic cattle commensal *E*. *coli*. Over three days at ambient laboratory temperature, *P*. *caudatum* consistently reduced both *E*. *coli* O157:H7 and non-Shiga toxigenic *E*. *coli* populations by 1–3 log cfu. Furthermore, a wild-type strain of Shiga-toxigenic *E*. *coli* O157:H7 (EDL933) and isogenic mutants lacking the A subunit of Stx 2a, the entire Stx 2a-encoding bacteriophage, and/or the entire Stx 1-encoding bacteriophage were grazed with similar efficacy by both *P*. *caudatum* and *Tetrahymena pyriformis* (another ciliate protozoon). Therefore, our data provided no evidence of a protective effect of either Stx or the products of other bacteriophage genes on protozoan predation of *E*. *coli*. Further research is necessary to determine if the grazing activity of naturally-occurring protozoa in cattle water troughs can serve to decrease cattle exposure to *E*. *coli* O157:H7 and other Shiga-toxigenic *E*. *coli*.

## Introduction

Diseases caused by the bacterium *Escherichia coli* O157:H7 are major public health concerns worldwide, with the highest numbers of reported cases in the United States, United Kingdom, Argentina, Canada and Japan [1, 2]. Many human infections are associated with the consumption of contaminated food and water, or through direct contact with cattle or their surrounding environments [3, 4]. *E*. *coli* O157:H7 virulence factors include the locus of enterocyte and effacement (LEE) pathogenicity island and one or more Shiga toxins (Stx), primarily Stx1, Stx2a, and Stx2c. The LEE pathogenicity island is responsible for attaching and effacing lesions seen in both humans and cattle. Stx are encoded by lambdoid lysogenic bacteriophages and are released from lysed bacteria. In humans, Stx targets endothelial cells in the colon contributing to hemorrhagic colitis [5], and renal glomerular endothelial cells in some cases resulting in the life-threatening disease, hemolytic uremic syndrome (HUS) [6, 7].

The high prevalence of carriage of Shiga toxin-encoding bacteriophages within the *E*. *coli* O157:H7 lineage raises a possibility that these prophages confer a selective advantage to the bacterium; however, the nature of this selective advantage is currently unknown. While Stx play a central role in *E*. *coli* O157:H7 pathogenesis in humans, the infection is likely too rare to provide the bacterium a significant selective advantage. In cattle, *E*. *coli* O157:H7 colonize the rectoanal junction (RAJ) resulting in transient and intermittent fecal shedding at levels as high as 10^6^ colony forming units (CFU) per gram of feces [8, 9]. Unlike humans, cattle lack vascular receptors for Stx [10] and therefore are not generally susceptible to disease associated with *E*. *coli* O157:H7 [10–12]. Furthermore, deletion of Stx genes does not change the ability of *E*. *coli* O157:H7 to colonize the bovine RAJ [13]. Given the lack of an observed selective role for Stx in mammalian hosts, it has been suggested that Stx may alternatively offer a selective advantage to *E*. *coli* O157:H7 in the environment by protection against predation (grazing) by free-living protozoa [14–17].

Previously, some investigators have observed that Stx-encoding bacteriophages protect *E*. *coli* O157:H7 from grazing by laboratory strains of *Tetrahymena pyriformis* [14] and *T*. *thermophila* [15, 17], even killing the latter [15, 17]. Other investigators have observed that *E*. *coli* O157:H7 is readily grazed by colpodean and oligohymenophorean ciliates [18, 19] isolated from dairy farm waste-water and by unidentified protozoa collected from cattle water troughs [20]. The goal of this study was to determine the roles of Stx and their encoding bacteriophages in protection against protozoan grazing. We evaluated the ability of protozoa to graze diverse genotypes of Shiga-toxigenic *E*. *coli* O157:H7 over three days using cattle water trough isolates of the protozoon, *Paramecium caudatum*. We also evaluated the ability of protozoa to graze *E*. *coli* O157:H7 strain EDL933 and isogenic mutants with deletion of the Stx2a subunit A-encoding nucleotide sequence, deletion of the entire Stx 2a-encoding bacteriophage sequence, and/or deletion of the entire Stx1-encoding bacteriophage sequence, using both *P*. *caudatum* and the laboratory strain of *T*. *pyriformis* previously described as susceptible to Stx [14].

## Materials and Methods

### Microbial agents

*Paramecium caudatum* was isolated from cattle water troughs where, frequently, it is a predominant member of the protozoan flora (unpublished observations). *P*. *caudatum* was cultivated in lidded Cellstar^®^ 24 well polystyrene culture plates with 1 ml of autoclaved trough water at ambient laboratory temperature, and the amplified protozoa were maintained at 4°C. Identification was confirmed by 18S rDNA sequences, as follows: Protozoan DNA was extracted (MagMAX^™^ Total Nucleic Acid Isolation Kit, AM1840, Applied Biosystems Inc.) per the manufacturer’s recommendations for liquid samples. Polymerase chain reaction (PCR) for the 18S rRNA gene was performed on the extracted DNA using primers (Invitrogen, Carlsbad, CA) P-SSU-342f and Medlin B described by Karnati et al [21] (Table 1). Each 25 μl PCR reaction contained 20 pmol of each primer, 1X PCR buffer, 125 μM of deoxynucleotide triphosphates (dNTP), 2mM magnesium chloride, and 2.5 U of Invitrogen^™^ Platinum Taq polymerase. Thermocycler (Bio-Rad iCycler) settings included an initial denaturation step (94°C, 2 minutes), followed by 35 cycles of [denaturation (94°C, 1 minute), annealing (37°C, 65 seconds), and extension (72°C, 3 minutes)] with a final 6 minute extension step at 72°C followed by a hold at 4°C. The 1,360-bp amplicon was visualized via gel electrophoresis, using a 0.8% agarose gel stained with ethidium bromide (Sigma-Aldrich, St. Louis, MO). The PCR product was cleaned using EXO/SAP with 8U Exonuclease 1 and 1.6 U FastAP, a Thermosensitive Alkaline Phosphatase (Thermo Scientific) at thermocycler settings of 20 minutes at 37°C, 15 minutes at 80°C, followed by a 4°C hold. The cleaned PCR product was submitted to Amplicon Express (Pullman, WA) for forward and reverse 18S sequencing using the same primers used for amplification. Sequences were compared to best fit matches using the NCBI BLAST algorithm (http://blast.ncbi.nlm.nih.gov/Blast.cgi).

**Table 1:** Sequence of primers for PCR amplification used throughout these studies.

Primer	Nucleotide Sequence	Objective	Ref.
P-SSU-342f	5′-CTTTCGATGGTAGTGTATTGGACTC-3 ′	Protozoa sequencing	[21]
Medlin B	5′-TGATCCTTCTGCAGGTTCACCTAC-3′	Protozoa sequencing	[24]
Stx2KOP1	5′-ATGAAGTGTATATTATTTAAATGGGTACTGTGCCTGTTACGTGTAGGCTGGAGCTGCTTC-3′	Stx 2a A subunit knock-out	[25]
Stx2KOP2	5′-TTATTTACCCGTTGTATATAAAAACTGTGACTTTCTGTTCCATATGAATATCCTCCTTAG-3′	Stx 2a A subunit knock-out	[25]
Stx1phageKO1	5′-TTACACAATTGGTGAAGTGGCGTTGCTTTGTGATATTAACGTGTAGGCTGGAGCTGCTTC-3′	Stx 1 phage knock-out	This work
Stx1phageKO2	5′-GTCTGTCCGTTGCGGTTTCAGCAATCCGTAACGCCTCTGCCATATGAATATCCTCCTTAG-3′	Stx 1 phage knock-out	This work
Stx2phageKO1	5′-GTTTATCATTTTGAAAAATATAATTTTATTTCATCCTCCTGTGTAGGCTGGAGCTGCTTC-3′	Stx 2a phage knock-out	This work
Stx2phageKO2	5′-AAACACAGAATTACCGAAGATCGTCCAAGAGCATATATTTCATATGAATATCCTCCTTAG-3′	Stx 2a phage knock-out	This work

*Tetrahymena pyriformis* strain GL-C, used as a control for bacterial grazing, was obtained from Cornell University (SD00707, *Tetrahymena* stock center, Ithaca, NY). *T*. *pyriformis* was cultivated at ambient laboratory temperature in 25cm^2^ polystyrene tissue culture flasks (Corning Glass Works, Corning, NY) in modified Neff medium (Rich Axenic Nutrient Media, https://tetrahymena.vet.cornell.edu/recipes.php) and amplified cultures were maintained at 4°C.

*E*. *coli* strains used in this study (Table 2) were maintained as glycerol stocks at -80°C. Overnight cultures of these bacterial stocks were prepared with the appropriate antibiotics (rifampicin 50 μg/ml, nalidixic acid 30 μg/ml, or chloramphenicol 25 μg/ml) according to their antibiotic resistance profile (Table 2) in Lennox broth (LB) (Invitrogen^™^, Carlsbad, CA) shaken (250 rpm) at 37°C. The overnight cultures were used to streak LB plates with the same selective antibiotics for isolation of colonies. Isolated colonies were subsequently grown as individual overnight cultures in LB without antibiotics for use in protozoa co-culture experiments and as inoculum for growth curves.

**Table 2:** *E*. *coli* strains used in these studies.

Strain	Parent strain source	Stx profile	Antibiotic resistance	Ref.
*E*. *coli* 186	Bovine feces	None	Rifampicin	[26, 27]
EDL933	Raw hamburger	Stx 1, Stx 2a	Nalidixic acid	[28]
E12056	Water, WA	Stx 2c	Nalidixic acid	[28]
E12058	Bovine feces, WA	Stx 1, Stx 2c	Nalidixic acid	[28]
E12061	Human clinical, WA	Stx 2a, Stx 2c	Nalidixic acid	[28]
E12064	Human clinical, WA	Stx 2a	Nalidixic acid	[28]
EDL933ΔStx 2a subunit A	Raw hamburger (EDL933)	Stx 1, Stx2aΔsubunit A	Chloramphenicol	This work
EDL933ΔStx 1 phage	Raw hamburger (EDL933)	Stx 2a	Chloramphenicol	This work
EDL933ΔStx 2a phage	Raw hamburger (EDL933)	Stx 1	Chloramphenicol	This work
EDL933ΔStx 1 & Stx 2a phages	Raw hamburger (EDL933)	None	Chloramphenicol	This work

### Preparation of *E*. *coli* O157:H7 isogenic mutants

The λ Red recombinase system was used to create isogenic mutants of *E*. *coli* O157:H7 EDL933. Mutants included deletions of: the gene encoding the A subunit of Stx 2a, the entire Stx 2a-encoding bacteriophage, the entire Stx 1-encoding bacteriophage, and both the Stx 1- and Stx 2a-encoding bacteriophages. Recombinant strains (Table 2) were created using the pKD46 and pKD3 plasmids (*E*. *coli* Genetic Resource Center, Yale University, New Haven, CT) as described by Datsenko et al. [22] and primers listed in Table 1. EDL933 was cultured in LB at 37°C shaking at 250 rpm to an optical density of 0.6 at 600 nm (OD_600_). The cells were made electrocompetent by washing three times with ice-cold 10% glycerol. Electrocompetent cells were transformed with the pKD46 plasmid via electroporation, and ampicillin (100μg/ml) was used to select for transformed colonies at 30°C overnight. Transformed colonies were grown in LB with 5mM L-arabinose at 30°C shaking at 200 rpm to an optical density of 0.6 at 600 nm (OD_600_). Cells were made electrocompetent as described above and transformed with the linear DNA fragment amplified from a pKD3 plasmid flanked by DNA sequences homologous to the knock-out target [22]. Chloramphenicol (25μg/ml) was used to select for transformed colonies at 30°C overnight. Isolated transformed colonies were then grown at 43°C to cure the pKD46 plasmid, and banked in glycerol at -80°C. Prior to banking, EDL933 mutants were confirmed using PCR for the knockout target gene (*stx2a* A subunit) and by Stx-associated bacteriophage insertion (SBI) genotyping for the Stx 2a-, Stx 1-, and combined Stx 1- & Stx 2a-bacteriophage knock-outs [23]. The loss of Stx production was confirmed by negative results on the Meridian ImmunoCard STAT!EHEC lateral flow devices (Bioscience, Inc., Germany) for Stx 1 and 2 compared with the parent strain.

### Protozoan grazing of Shiga-toxigenic *E*. *coli* compared to non-Shiga toxigenic *E*. *coli*

Protozoa culture suspensions (1 ml) were added to 1.5 ml autoclaved conical tubes with loosened caps (USA Scientific). On day zero, Shiga toxigenic *E*. *coli* and non-Shiga toxigenic *E*. *coli* with selectable resistance markers (Table 2) were added to these suspensions to create mixed cultures, with multiplicities of infection (MOI) between 1 x 10^4^ and 1 x 10^5^ bacterial cfu per protozoan. Colony forming units were counted from serial decimal dilutions of the co-culture suspensions following 0, 24, 48 and 72 hours of co-culture using LB plates with appropriate selective antibiotics. Protozoan densities were determined every 24 hours via light microscopy. For each co-culture experiment, parallel cultures containing the same bacterial inoculum in the absence of protozoa served as negative controls, using sterile modified Neff media for *T*. *pyriformis* assays and cattle trough water treated with 10 μg/ml cycloheximide (Sigma-Aldrich, St. Louis, MO) for *P*. *caudatum* assays. Three biological replicates were performed for all co-culture experiments.

### Growth patterns of *E*. *coli* O157:H7, cattle commensal *E*. *coli*, and EDL933 mutants

Bacterial growth curves for each of the bacterial strains (Table 2) were determined using a Bioscreen C MBR (Oy Growth Curves AB Ltd., Piscataway, NJ). For each growth curve, approximately 1 x 10^7^ bacteria/ml of individual bacterial strains used throughout the study was added to ten separate wells (five biological replicates) of a sterilized Honeycomb 2 100-well plate (Oy Growth Curves AB Ltd.). Bacterial inocula were suspended in cycloheximide-treated trough water (*P*. *caudatum* studies) or modified Neff media (*T*. *pyriformis* studies) to mimic the culture conditions of co-culture experiments. The mean OD_420-580nm_ (wideband) were determined every 2 hours for three days at ambient laboratory temperature (23°C) without shaking to create growth curves (S1 and S2 Figs).

### Statistical analysis

Repeated measures ANOVAs were used to evaluate differences in protozoan grazing of Shiga-toxigenic and non-Shiga toxigenic *E*. *coli* strains over three days of co-culture and in protozoan proliferation over three days with and without added *E*. *coli*. *P*-values <0.05 were considered statistically significant.

## Results

### *P*. *caudatum* grazing of *E*. *coli* O157:H7

**Fig 1:**
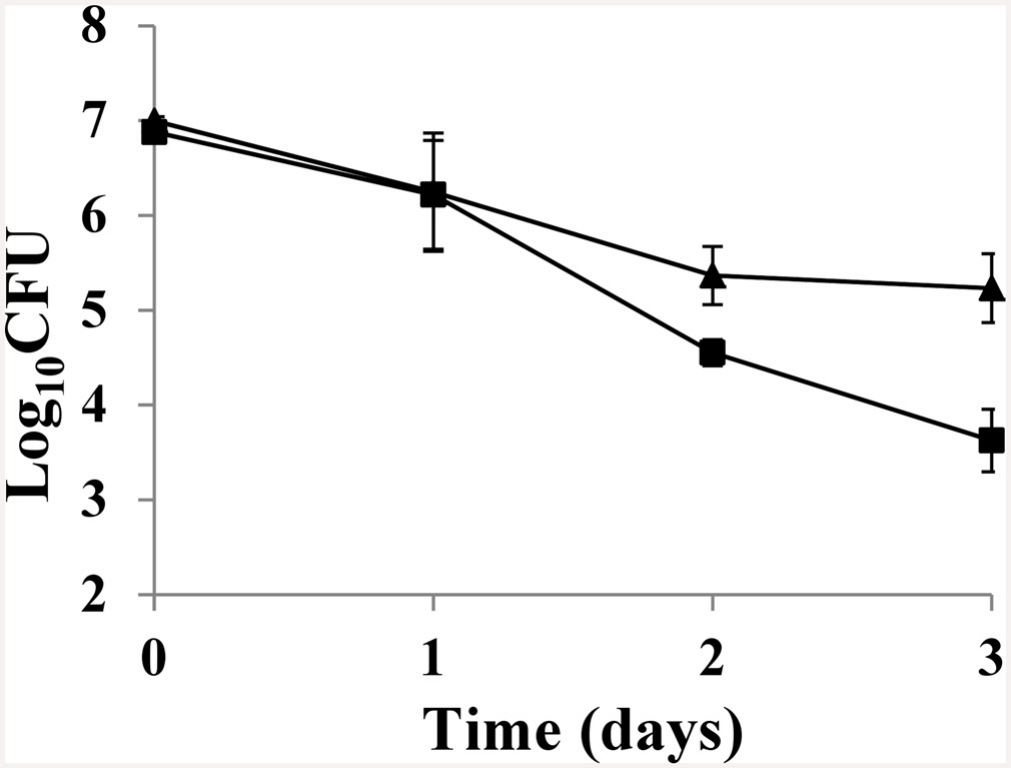
*P*. *caudatum* grazing of *E*. *coli* O157:H7 EDL933 and cattle commensal *E*. *coli* (186) over three days at ambient laboratory temperature. CFU (mean ± s. e.) for three grazing trials each containing a matched MOI of *E*. *coli* O157:H7 EDL933 (squares) and cattle commensal *E*. *coli* (triangles).

**Fig 2:**
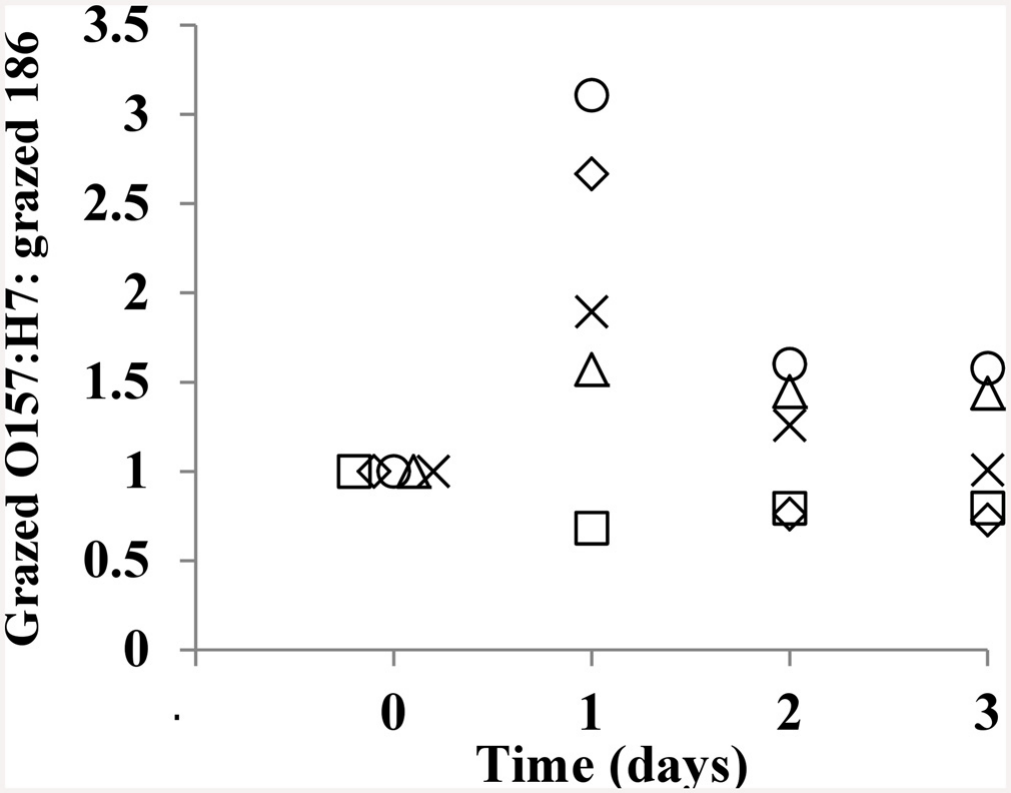
Ratios of CFU of five strains of *E*. *coli* O157:H7 compared to matched MOI of cattle commensal *E*. *coli* (186) grazed by *P*. *caudatum* over three days at ambient laboratory temperature. Five strains of *E*. *coli* O157:H7 (Table 2) containing different Shiga toxin profiles: EDL933 (squares), E12056 (diamonds), E12058 (circles), E12061 (triangles), E12064 (X).

**Fig 3:**
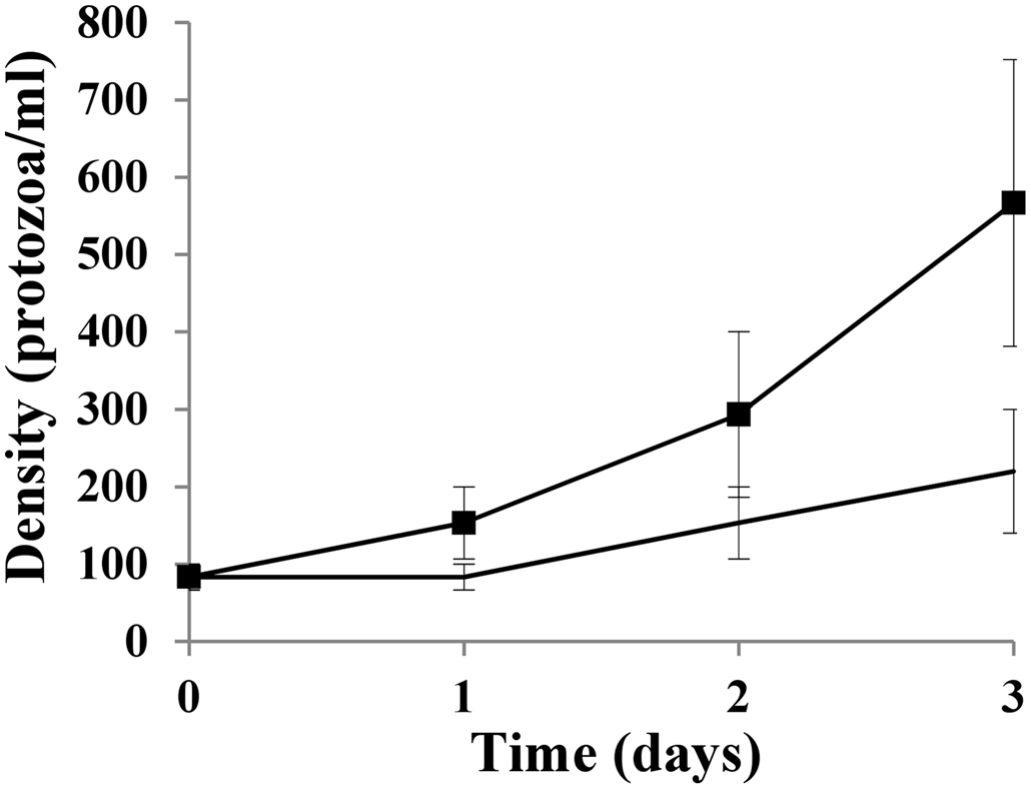
Density (mean ± s. e.) of *P*. *caudatum* when co-cultured with matched MOI of *E*. *coli* O157:H7 EDL933 and cattle commensal *E*. *coli* (186) for three days at ambient laboratory temperature. Squares, *P*. *caudatum* with added *E*. *coli*; no marker, *P*. *caudatum* in trough water without added bacteria.

When co-cultured with *P*. *caudatum* for 3 days, viable cell counts of both Shiga-toxigenic *E*. *coli* O157:H7 strain EDL933 and cattle commensal Stx-negative *E*. *coli* strain #186 were reduced by 1–3 log cfu/ml (Fig 1). No statistically significant differences in the reduction of Shiga-toxigenic versus non-Shiga toxigenic *E*. *coli* strains were observed. Fig 2 depicts the ratios of protozoan-grazed Shiga-toxigenic *E*. *coli* to grazed non-Shiga toxigenic *E*. *coli*. Five diverse strains of Shiga-toxigenic *E*. *coli*, each with a different Stx profile (Table 2), were used in these co-culture experiments along with cattle commensal *E*. *coli* strain #186. While after the first day there was some variation in the ratios of Shiga-toxigenic:non-Shiga toxigenic bacteria grazed by *P*. *caudatum*, by the end of the second and third days, the ratios were near unity for all strains used in this study, with no apparent effect by Stx profile. No decrease in the numbers of *P*. *caudatum* were observed for over the three day period; the tendency was instead for increased protozoan numbers compared to control cultures without added bacteria (Fig 3).

### Comparison of wild-type Shiga-toxigenic *E*. *coli* O157:H7 strain EDL933 with *stx*-negative and Stx-encoding bacteriophage negative isogenic mutants grazed by protozoa

**Fig 4:**
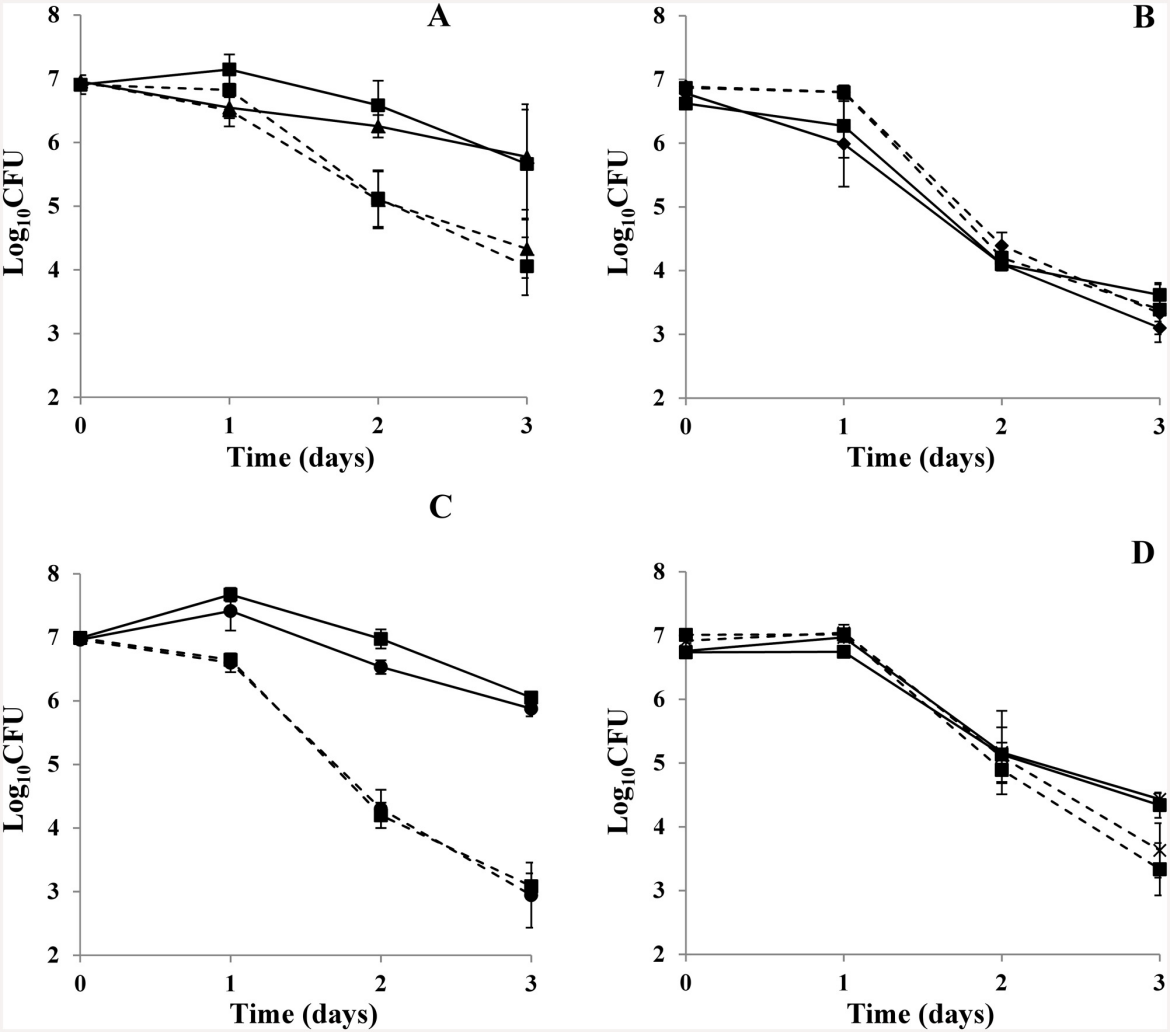
CFU (mean ± s. e.) of *E*. *coli* O157:H7 EDL933 and isogenic mutants inoculated at matched MOI when co-cultured with *T*. *pyriformis* (solid lines) and *P*. *caudatum* (dashed lines) at ambient laboratory temperature. (A) *E*. *coli* O157:H7 EDL933 (squares) and EDL933ΔStx 2a subunit A (triangles). (B) *E*. *coli* O157:H7 EDL933 (squares) and EDL933ΔStx 1 phage (diamonds). (C) *E*. *coli* O157:H7 EDL933 (squares) and EDL933ΔStx 2a phage (circles). (D) *E*. *coli* O157:H7 EDL933 (squares) and EDL933ΔStx 1 & Stx 2a phages (asterisks).

*P*. *caudatum* grazed the wild-type strain of *E*. *coli* O157:H7 (EDL933), *stx2a*-negative EDL933, and EDL933 completely lacking the Stx 1-, Stx 2-, and the Stx 1- and Stx 2-encoding bacteriophages at very similar degrees, reducing the mutant strains and the wild-type strain by approximately 3-logs over three days (Fig 4), with a tendency towards greater predation of the wild-type strain compared to any of the Stx mutants by the end of three days. Co-cultures of the wild-type and mutant strains of EDL933 with *T*. *pyriformis* produced very similar reductions of 1- to 3-logs, and no statistically significant differences in the degree of grazing of these strains were observed at any time point. In contrast to *P*. *caudatum*, at the end of day 1 of co-culture with *T*. *pyriformis* there is a trend towards greater predation of mutants compared to wild-type *E*. *coli* O157:H7. However, this trend disappeared by the second day. Similar to *P*. *caudatum*, *T*. *pyriformis* also proliferated over three days when co-cultured with Shiga-toxigenic *E*. *coli* (data not shown).

## Discussion

In contrast to previous reports that carriage of Shiga toxin-encoding bacteriophages by *E*. *coli* O157:H7 provides protection against grazing protozoa [14–17], our experiments did not detect a protective effect of Stx expression or by other factors encoded on the Stx-encoding bacteriophages of *E*. *coli* O157:H7 against grazing protozoa. In Fig 4, within the first 24 hours, there was a trend for higher viable cell counts of wild-type *E*. *coli* O157:H7 compared to the Stx mutants when co-cultured with *T*. *pyriformis*, similar to previous reports with *T*. *thermophila* [15, 17]. However, the transient difference was not statistically significant and the trend was not seen with co-cultured *P*. *caudatum*. In fact, by the end of the third day, the trend was opposite for *P*. *caudatum*, with more of the wild-type *E*. *coli* O157:H7 grazed than the Stx mutants. Overall, the variation in grazing of wild-type *E*. *coli* O157:H7 compared to Stx mutants by *P*. *caudatum* or *T*. *pyriformis* was not statistically significant at any time point throughout the course of the study and protection against grazing by the Stx genes or prophage was not demonstrated.

There was also no statistically significant difference when comparing the ratios of five different strains of grazed *E*. *coli* O157:H7 to cattle commensal *E*. *coli*, with the ratios approaching unity for all strains by the second day and remaining near unity by the end of the study. Each strain of *E*. *coli* O157:H7 had a different Stx profile, with none of the different Stx profiles providing protection against grazing protozoa. While the reason(s) underlying the differences between our results and some previously published findings [14–17] is unknown, potentially the different strains of *E*. *coli* and protozoa used in this study and the different (longer) time course investigated may have played a role. In contrast, the results of this study are consistent with the findings of others [18–20] that at least some environmental protozoa on cattle farms efficiently graze *E*. *coli* O157:H7. Further investigation of the conditions under which Stx expression may protect against protozoan predation is required to clarify these observations.

Several species of protozoa graze bacteria as a food source. *P*. *caudatum* is a free-living ciliate protozoon commonly found in cattle farm water troughs. This species is highly bactivorous as demonstrated in this report, similar in this regard to *T*. *pyriformis*, another free-living ciliate that has been frequently used in biomedical research. While other environmental strains of protozoa may have preferences on bacterial predation, no such preferences were observed with either of these species in regard to Shiga-toxigenic *E*. *coli* compared to non-Shiga toxigenic *E*. *coli*.

The proliferation of both *T*. *pyriformis* and *P*. *caudatum* in the presence of Shiga-toxigenic *E*. *coli* demonstrates the ability of these protozoa to use the bacteria as a food source for growth and replication. In contrast to the reports evaluating *Tetrahymena thermophila* [15, 17] and *Acanthamoeba castellanii* [16], there was no indication that either *T*. *pyriformis* or *P*. *caudatum* was killed by Shiga-toxigenic *E*. *coli* O157:H7.

The growth and survival of cattle commensal *E*. *coli*, wild-type EDL933 and Stx mutants were similar in cycloheximide-treated trough water over three days (S1 Fig). Within the first 24 hours, there was increased optical density of cattle commensal *E*. *coli* at a few time points, however, this slight variation was not seen for the remainder of the study (S1 Fig inset). In these studies, neither Stx nor their encoding bacteriophages provided a selective advantage for *E*. *coli* O157:H7. In modified Neff media, optical density measurements suggested that the bacteriophage knock-out mutant strains reached stationary phase at lower numbers compared to wild-type *E*. *coli* O157:H7 EDL933 (S2 Fig). This observation does not change the conclusion that protozoa graze Shiga-toxigenic *E*. *coli* as well as non-Shiga toxigenic *E*. *coli*; rather increased grazing of Shiga-toxigenic *E*. *coli* would be required if the wild type strains proliferate to higher levels than the mutants.

The use of several isogenic mutants of *E*. *coli* O157:H7 and the use of a common environmental protozoon isolated from farms in the inland Northwest (*P*. *caudatum*) differentiates the results of this study from previous literature. This report provides strong evidence that the *stx* genes and Stx-encoding bacteriophages do not protect *E*. *coli* O157:H7 against grazing by some of the most frequently occurring strains of environmental protozoa in cattle water troughs. Continued research is necessary to investigate other potential roles of Shiga toxins and their encoding bacteriophages in the environment and in the cattle host. Additionally, since commonly-occurring *P*. *caudatum* are excellent grazers of *E*. *coli* O157:H7, evaluating the use of these protozoa as a pre-harvest control measure on cattle farms is also warranted.

## Supporting Information

S1 Fig Growth curves of *E*. *coli* O157:H7, cattle commensal *E*. *coli* (186) and Shiga toxin mutants in cycloheximide-treated trough water. Optical density measurements at wideband wavelength (420–580 nm) over three days at ambient laboratory temperature (23°C). No marker, cycloheximide-treated water without added bacteria; squares, *E*. *coli* O157:H7 EDL933; triangles, cattle commensal *E*. *coli* (186); circles, EDL933ΔStx 1 phage; diamonds, EDL933ΔStx 2a subunit A; X, EDL933ΔStx 2a phage; plus, EDL933ΔStx 1 and Stx 2a phages. By the end of the first 24 hours, 95% confidence interval bars on *E*. *coli* O157:H7 EDL933 overlap with 186 and all isogenic mutants for the remainder of the study (inset).
(TIFF)

S2 Fig Growth curves of *E*. *coli* O157:H7 and Shiga toxin mutants in modified Neff media. Optical density measurements at wideband wavelength (420–580 nm) over three days at ambient laboratory temperature (23°C). No marker, modified Neff media without added bacteria; squares, *E*. *coli* O157:H7 EDL933; circles, EDL933ΔStx 1 phage; diamonds, EDL933ΔStx 2a subunit A; X, EDL933ΔStx 2a phage; plus, EDL933ΔStx 1 and Stx 2a phages. 95% confidence interval bars on *E*. *coli* O157:H7 EDL933 overlap with EDL933ΔStx 2a subunit A. All bacteriophage knock-out mutants reach a lower optical density by stationary phase and for the remainder of the growth curves, the optical densities of the knock-out mutants missing at least one entire bacteriophage do not overlap with the 95% confidence interval bars on *E*. *coli* O157:H7 EDL933.
(TIFF)

## References

[1] Irino K, Ibelli Vaz TM, Kato M, Naves Z, Lara RR, Carvalho Marco ME, et al (2002) O157:H7 Shiga Toxin-Producing *Escherichia coli* Strains Associated with Sporadic Cases of Diarrhea in São Paulo, Brazil. Emerg Infect Dis 8: 446–447. 11971785 10.3201/eid0804.010490 PMC2730246

[2] Griffin PM, Tauxe RV. (1991) The epidemiology of infections caused by *Escherichia coli* O157:H7, other enterohemorrhagic *E*. *coli*, and the associated hemolytic uremic syndrome. Epidemiol Rev 13: 60–98. 1765120 10.1093/oxfordjournals.epirev.a036079

[3] Armstrong GL, Hollingsworth J, Morris JG. (1996) Emerging foodborne pathogens: *Escherichia coli* O157:H7 as a model of entry of a new pathogen into the food supply of the developed world. Epidemiol Rev 18: 24–51. 10.1093/oxfordjournals.epirev.a017914 8877329

[4] Sargeant JM, Amezcua MR, Rajic A, Waddell L. (2007) Pre-harvest interventions to reduce the shedding of *E*. *coli* O157 in the faeces of weaned domestic ruminants: a systematic review. Zoonoses Public Hlth 54: 260–277. 10.1111/j.1863-2378.2007.01059.x 17803515

[5] Kelly JK, Oryshak A, Wenetsek M, Grabiec J, Handy S. (1990) The colonic pathology of *Escherichia coli* O157:H7 infection. Am J Surg Pathol 14: 87–92. 2403759 10.1097/00000478-199001000-00010

[6] Richardson SE, Karmali MA, Becker LE, Smith CR. (1988) The histopathology of the hemolytic uremic syndrome associated with verocytotoxin-producing *Escherichia coli* infections. Hum Pathol 19: 1102–1108. 3047052 10.1016/s0046-8177(88)80093-5

[7] Zoja C, Buelli S, Morigi M. (2010) Shiga toxin-associated hemolytic uremic syndrome: pathophysiology of endothelial dysfunction. Pediatr Nephrol 25: 2231–2240. 10.1007/s00467-010-1522-1 20424866

[8] Besser TE, Richards BL, Rice DH, Hancock DD. (2001) *Escherichia coli* O157:H7 infection in calves: infectious dose and direct contact transmission. Epidemiol Infect 127: 555–560. 11811890 10.1017/s095026880100615x PMC2869782

[9] Mechie SC, Chapman PA, Siddons CA. (1997) A fifteen month study of *Escherichia coli* O157:H7 in a dairy herd. Epidemiol Infect 118: 17–25. 9042031 10.1017/s0950268896007194 PMC2808768

[10] Pruimboom-Brees IM, Morgan TW, Ackermann MR, Nystrom ED, Samuel JE, Cornick NA, et al 2000 Cattle lack vascular receptors for *Escherichia coli* O157:H7 Shiga toxins. PNAS 97: 10325–10329. 10973498 10.1073/pnas.190329997 PMC27023

[11] Cray WC Jr., Moon HW. (1995) Experimental infection of calves and adult cattle with *Escherichia coli* O157:H7. Appl Environ Microbiol 61: 1586–1590. 7747972 10.1128/aem.61.4.1586-1590.1995 PMC167413

[12] Brown CA, Harmon BG, Zhao T, Doyle MP. (1997) Experimental *Escherichia coli* O157:H7 carriage in calves. Appl Environ Microbiol 63: 27–32. 8979335 10.1128/aem.63.1.27-32.1997 PMC168298

[13] Sheng H, Lim JY, Knecht HJ, Li J, Hovde CJ. (2006) Role of *Escherichia coli* O157:H7 Virulence Factors in Colonization at the Bovine Terminal Rectal Mucosa. Infect Immun 74: 4685–4693. 16861656 10.1128/IAI.00406-06 PMC1539576

[14] Steinberg KM, Levin BR. (2007) Grazing protozoa and the evolution of the *Escherichia coli* O157:H7 shiga toxin-encoding prophage. Proc R Soc B 274: 1921–1929. 17535798 10.1098/rspb.2007.0245 PMC2211389

[15] Lainhart W, Stolfa G, Koudelka GB. (2009) Shiga toxin as a bacterial defense against a eukaryotic predator, *Tetrahymena thermophila*. J Bacteriol 191: 5116–5122. 10.1128/JB.00508-09 19502393 PMC2725575

[16] Arnold JW, Koudelka GB. (2014) The Trojan Horse of the microbiological arms race: phage-encoded toxins as a defense against eukaryotic predators. Environ Microbiol 16: 454–466. 10.1111/1462-2920.12232 23981100

[17] Stolfa G, Koudelka GB. (2012) Entry and killing of *Tetrahymena thermophila* by bacterially produced Shiga toxin. mBio, 4:e00416–12. 10.1128/mBio.00416-12 23269826 PMC3531803

[18] Ravva SV, Sarreal CZ, Mandrell RE. (2013). Altered protozoan and bacterial communities and survival of *Escherichia* coli O157:H7 in monensin-treated wastewater from a dairy lagoon. PLosOne 8:e54782 10.1371/journal.pone.0054782 PMC3551901 23349969

[19] Ravva SV, Sarreal CZ, Mandrell RE. (2010) Identification of protozoa in dairy lagoon wastewater that consume *Escherichia* coli O157:H7 preferentially. PlosOne 5:e15671 10.1371/journal.pone.0015671 PMC3004959 21187934

[20] LeJeune JT, Besser TE, Merrill NL, Rice DH, Hancock DD. (2001) Livestock drinking water microbiology and the factors influencing the quality of drinking water offered to cattle. J Dairy Sci 84: 1856–1862. 11518311 10.3168/jds.S0022-0302(01)74626-7

[21] Karnati SR, Yu Z, Sylvester T, Dehority BA, Morrison M, Firkins JL. (2003) Technical note: Specific PCR amplification of protozoal 18S rDNA sequences from DNA extracted from ruminal samples of cows. J Anim Sci 81: 812–815. 12661662 10.2527/2003.813812x

[22] Datsenko KA, Wanner BL. (2000) One-step inactivation of chromosomal genes in *Escherichia coli* K-12 using PCR products. PNAS 97: 6640–6645. 10829079 10.1073/pnas.120163297 PMC18686

[23] Shringi S, Schmidt CE, Kaya K, Brayton KA, Hancock DD, Besser TE. (2012) Carriage of stx2a differentiates clinical and bovine-biased strains of *Escherichia* coli O157. PLoS ONE 7:e51572 10.1371/journal.pone.0051572 23240045 PMC3519850

[24] Medlin L, Elwood HJ, Stickel S, Sogin ML. (1988) The characterization of enzymatically amplified eukaryotic 16S-like rRNA-coding regions. Gene 71: 491–499. 3224833 10.1016/0378-1119(88)90066-2

[25] Ho NK, Ossa JC, Silphaduang U, Johnson R, Johnson-Henry KC, Sherman PM. (2012) Enterohemorrhagic *Escherichia coli* O157:H7 Shiga toxins inhibit gamma interferon-mediated cellular activation. Infect Immun 80: 2307–2315. 10.1128/IAI.00255-12 22526675 PMC3416474

[26] Sawant AA, Casavant NC, Call DR, Besser TE. (2011) Proximity- dependent inhibition in *Escherichia coli* isolates from cattle. Appl Environ Microbiol 77: 2345–2351. 10.1128/AEM.03150-09 21296941 PMC3067425

[27] Eberhart LJ, Deringer JR, Brayton KA, Sawant AA, Besser TE, Call DR. (2012) Characterization of a novel microcin that kills enterohemorrhagic *Escherichia coli* O157:H7 and O26. Appl Environ Microbiol 78: 6592–6599. 10.1128/AEM.01067-12 22773653 PMC3426703

[28] Shringi S, Garcia A, Lahmers KK, Potter KA, Muthupalani S, Swennes AG, et al (2012) Differential virulence of clinical and bovine-biased enterohemorrhagic *Escherichia coli* O157:H7 genotypes in piglet and Dutch belted rabbit models. Infect Immun 80: 369–380. 10.1128/IAI.05470-11 22025512 PMC3255674

