# Supplementary figures and images for: Protozoan Predation of Escherichia coli O157:H7 Is Unaffected by the Carriage of Shiga Toxin-Encoding Bacteriophages
Source: PLoS One. 2016 Jan 29;11(1):e0147270. doi: 10.1371/journal.pone.0147270 (PMC4732659; doi:10.1371/journal.pone.0147270)

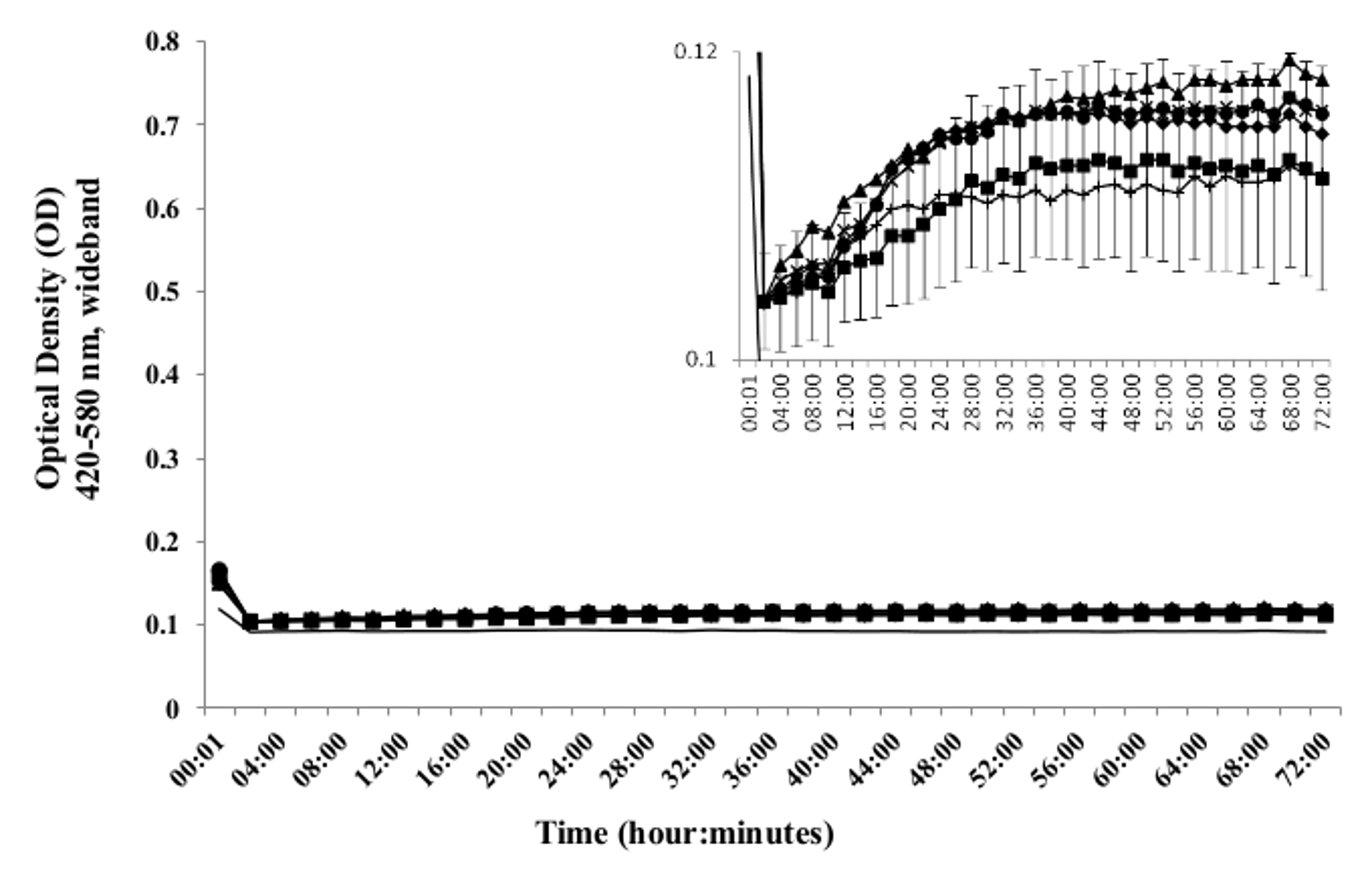

Supplement: S1 Fig — Optical density measurements at wideband wavelength (420–580 nm) over three days at ambient laboratory temperature (23°C). No marker, cycloheximide-treated water without added bacteria; squares, E. coli O157:H7 EDL933; triangles, cattle commensal E. coli (186); circles, EDL933ΔStx 1 phage; diamonds, EDL933ΔStx 2a subunit A; X, EDL933ΔStx 2a phage; plus, EDL933ΔStx 1 and Stx 2a phages. By the end of the first 24 hours, 95% confidence interval bars on E. coli O157:H7 EDL933 overlap with 186 and all isogenic mutants for the remainder of the study (inset). (TIFF) [file pone.0147270.s001.TIFF]

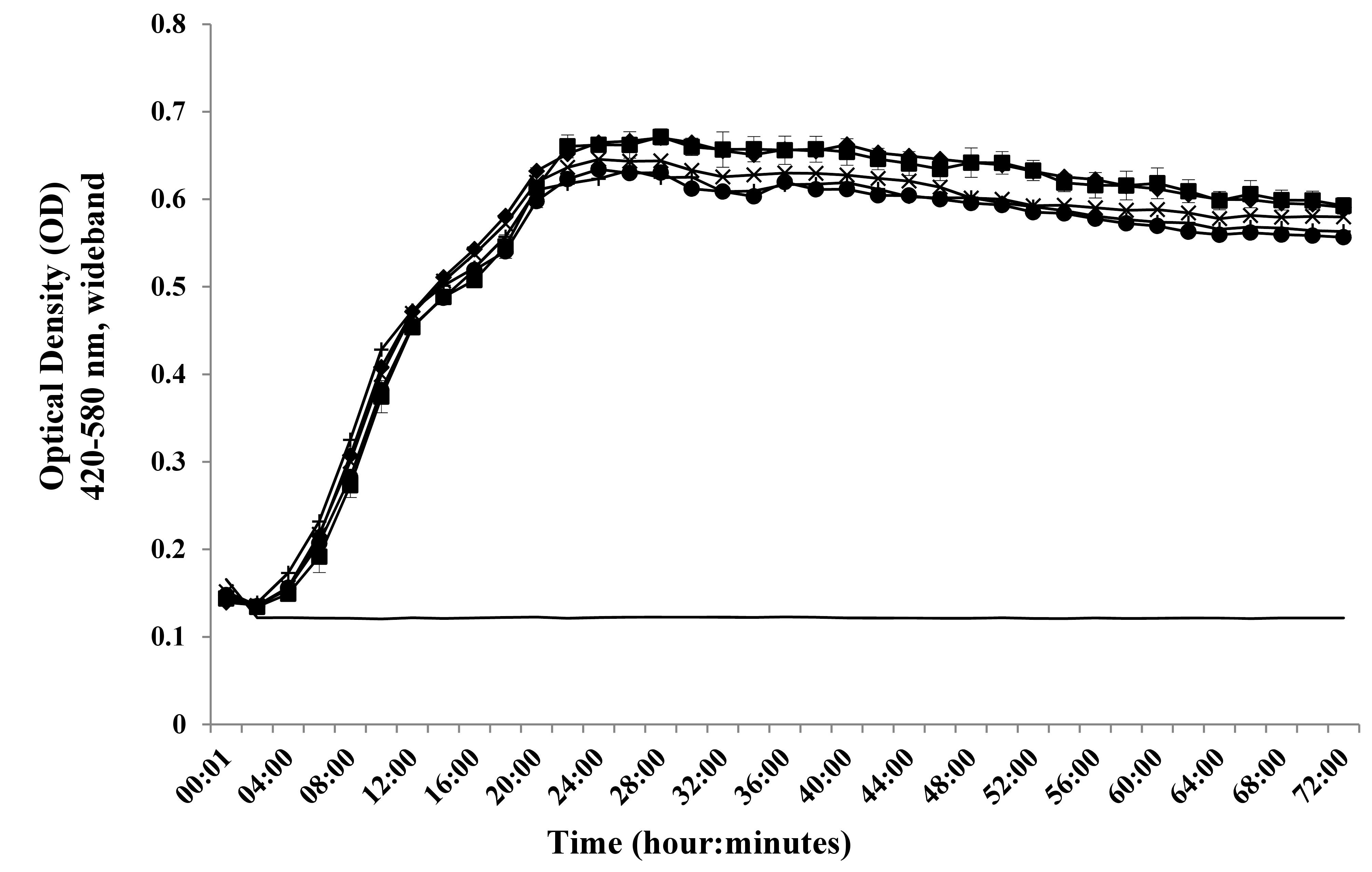

Supplement: S2 Fig — Optical density measurements at wideband wavelength (420–580 nm) over three days at ambient laboratory temperature (23°C). No marker, modified Neff media without added bacteria; squares, E. coli O157:H7 EDL933; circles, EDL933ΔStx 1 phage; diamonds, EDL933ΔStx 2a subunit A; X, EDL933ΔStx 2a phage; plus, EDL933ΔStx 1 and Stx 2a phages. 95% confidence interval bars on E. coli O157:H7 EDL933 overlap with EDL933ΔStx 2a subunit A. All bacteriophage knock-out mutants reach a lower optical density by stationary phase and for the remainder of the growth curves, the optical densities of the knock-out mutants missing at least one entire bacteriophage do not overlap with the 95% confidence interval bars on E. coli O157:H7 EDL933. (TIFF) [file pone.0147270.s002.TIFF]
